# My Problems Are Solvable: Idiographic Methods Offset Age Differences in Interpersonal Problem Solving Among Young, Middle-Aged, and Older Adults

**DOI:** 10.3389/fpsyg.2019.00276

**Published:** 2019-02-12

**Authors:** Daniele Artistico, Daniel Cervone, Carolina Montes Garcia

**Affiliations:** ^1^Baruch College, The City University of New York, New York City, NY, United States; ^2^Department of Psychology, University of Illinois at Chicago, Chicago, IL, United States; ^3^Research Experiences for Undergraduates (REU), Baruch College, Columbia University, New York City, NY, United States

**Keywords:** everyday problem solving, idiographic methods, older adults, younger adults, middle-aged adults

## Abstract

This study tested the hypothesis that older adults retain high levels of everyday problem solving performance when confronting problems of maximal ecological relevance, identified through idiographic methods. Younger, middle-aged, and older adults completed a daily challenge questionnaire (DCQ) in which they reported problems of maximal personal relevance or idiographic problems. The large majority of the problems reported were interpersonal. We then assessed performance on an everyday problem-solving task in which participants generated solutions for idiographic problems as well as problems generated by group matched research participants representing each of two other age groups (e.g., older adults received their own problems plus problems generated by matched younger and middle-aged adults). Performance was measured by computing the total number of safe and effective solutions provided. Results fully supported our hypothesis; adults of all ages showed higher performance when solving their idiographic problems.

## Introduction

How does cognition change across the course of life? On the one hand, a number of basic information-processing capacities – perceptual abilities, the control of attention, and working memory – decline inevitably with advancing age (Birren and Schaie, 2005; Gaesser et al., 2017). Yet, age brings social experiences that elaborate on and enrich the mental representation that provides the basis of both self-knowledge and social skills (Carstensen et al., 2011). Older adults may thus gain pragmatic wisdom in handling the challenges of life (Baltes and Staudinger, 2000) that help offset losses in speed at the information processing level.

A cognitive task that has proven to be revealing of such age-related trends is everyday problem solving, defined as the solving of life challenges that generally are open to more than one solution (Mienaltowski, 2011). The capacity to generate solutions to everyday problems rests partly on the ability to draw upon mentally represented and socially acquired knowledge. Everyday problems may thus be a domain in which older adults maintain high levels of performance despite experiencing information-processing declines.

Evidence on this point, however, is somewhat mixed. On overall indices of everyday problem solving performance, younger adults outperform older adults (Thornton and Dumke, 2005). Moreover, in one study, older and younger participants were asked to rate several everyday problem solving questions. These questions were about personal salience, general salience, age-appropriateness, and how often they have encountered such a problem in their own lives. Importantly, these factors were not found to offset age differences in everyday problem solving (Thornton et al., 2013). Also, middle-aged adults’ performance on everyday problems involving interpersonal conflicts or family emergencies is typically superior to that of other age groups (Denney et al., 1982; Denney and Pearce, 1989).

Yet, a number of considerations suggest that older adults may perform as well as younger adults in select domains (see Artistico et al., 2010). As Strough and Keener (2014) explain, the possibility of domain-linked variations in performance is heightened by taking a contextualist perspective on everyday problem solving. A contextualist perspective (e.g., Berg et al., 1998a) highlights the ways in which an individual’s coping strategies and task appraisals develop within, and thus are inherently linked to, distinct domains that comprise the individual’s life (cf. Lazarus, 1991; Shoda et al., 2007; Cervone et al., 2010). This perspective indicates that different domains may trigger variations in cognitive processing that, in turn, foster within-person, and across-context variation in performance. For example, problem-solving skills are impaired when “surface features” (circumstances described in a problem statement that are not critical to its solution) prevent people from seeing “deep structure” that, if grasped would enable them to devise problem solutions (Chi and VanLehn, 2012). If so, older adults may display relatively high everyday problem solving performance in familiar domains in which confusing or distracting surface are absent. A challenging yet familiar situation – for example, a recurring interpersonal problem – thus may be a select domain within which a given older adult may be able to generate numerous viable problem solutions.

A more general consideration is grounded in analyses of intraindividual variation in experience and behavior across social contexts (e.g., Mischel and Shoda, 1995). For example, Ram and Gerstorf’s (2009) approach to the dynamics of development, which highlights the significance of studying development in contexts of relevance to the individual, indirectly suggests that older adults may maintain problem-solving abilities in select domains. This perspective aligns research and theory indicating that older adults remain highly adaptive in settings that are important to them (Cornelius and Caspi, 1987; Baltes, 1997; Blanchard-Fields, 2007).

However, such general frameworks do not answer the question of which domains, exactly, are the ones in which problem-solving performance may be maintained. How can those domains be identified? Specifically, if older adults maintain domain-linked expertise, how can one identify the domains?

### Age-Group-Centered Analyses

One strategy for identifying key life domains is an *age-group-centered strategy*. Older adults may, on average, perform at higher levels when confronting a class of problems that is generally characteristic of the lives of the typical member of their age group. Among younger adults, the familiarity of their age-group-relevant problems may trigger positive *in-(age)group* reinforcement (Dowd and Artistico, 2016) that enhances performance - or may serve as a subtle cue that raises self-efficacy appraisal, which, in turn, increase motivation and task persistence (cf. Cervone and Peake, 1986). These possibilities are also consistent with Blanchard-Field’s Blanchard-Fields (2007) analysis of how older adults adjust their cognitive strategies in response to contextual cues (i.e., eye contact) especially since she underlined how such cues are more meaningful in interpersonal domains (i.e., resolving a conflict with a friend) rather then instrumental domains (i.e., maintenance and repairs at one’s home).

Empirical findings on the influence of age-appropriate contexts, however, is somewhat mixed. Artistico et al. (2003) found that older adults displayed levels of perceived self-efficacy and everyday problem-solving performance that exceeded those of younger adults when confronted with age-appropriate problems. Similarly, Artistico et al. (2010) reported that when problems are framed in terms of the lives of older adults, older adults outperform other age groups. However, in a report by Thornton et al. (2013), older adults did not display superior performance on age-appropriate problems, even when those problems contained interpersonal content that had been expected to maximize older-adult performance.

The mixed nature of these results could be interpreted as indicating that older adults do not robustly maintain the ability to solve everyday problems. An alternative possibility, however, is that the methodology – specifically, the use of generically age-appropriate problem sets – may be inadequate to reveal older adults’ maximal problem-solving abilities. For example, Thornton et al. (2013) examined problem-solving performance on each of four categories of problems that varied in content (practical versus interpersonal) and age relevance (age-neutral vs. older-age). All participants attempted to solve common sets of problems of these various types. Thornton et al. (2013) found that older-adult performance was not superior in contexts that, in general, were more relevant to older-adult life.

The distinctive question that we pursue is whether older-adult performance may be superior when problem domains are identified idiographically, rather than through age-group-relevant methods. Idiographic methods have a long tradition in psychological science (Allport, 1937; Cantor and Kihlstrom, 1987; Caprara and Cervone, 2000) but to our knowledge, they have not been applied to everyday problem-solving ability. Unlike previous methodology (cf. Blanchard-Fields et al., 1997; Allaire and Marsiske, 2002; Artistico et al., 2003, 2010; Blanchard-Fields et al., 2007; Thornton et al., 2013), we employed idiographic procedures in our current study, in which participants attempted some problems in domains that were distinctively relevant to themselves. Specifically, they solved self-reported everyday problems that were labeled “own problems.”

The virtues of idiographic methods inevitably are accompanied by costs. In any study, idiographic methods maximize the personal relevance of stimulus material while simultaneously entailing a sacrifice of experimental control. In everyday problem-solving research, loss of experimental control occurs in that, when different age groups identify personally-relevant problems through idiographic methods, the groups’ problem sets may vary on a number of different dimensions. In the present study, we chose to accept this relevance—control trade-off. In other words, in light of the relative lack of prior research employing such a strategy, we deliberatively decided to explore problem-solving performance in domains of maximal relevance to the individual, as identified through idiographic methods.

We expect within subject differences for solving problems of their own versus others’ problems across all age groups. We also predict an interaction type of problem and age group. Specifically, we hypothesize that younger adults would do better than middle-aged and older adults on solving younger adults problems, middle-aged adults would do better than younger and older adults on solving middle-aged adult problems, and older adults would do better than younger and middle-aged adults in solving older adult problems.

## Materials and Methods

### Participants

Participants were recruited through newspaper advertisements, public postings, and personal contact in recreation centers. To ensure that the study sample represented the diverse urban Chicago area in which the research was conducted, recruiting efforts substantially targeted publications and institutions serving ethnic and racial minorities. All participants signed a consent form document. The Institutional Review Board of the University of Illinois at Chicago approved the study procedures.

Of 114 individuals who responded to recruitment, 85^1^ completed all study procedures. In this group, 62% were female, 87% were native speakers of English, all were literate (however, for those who preferred oral rather than written study procedures, that option was made available), and all possessed at least a high school degree. The sample was 47% White, 21% Black, 20% Asian 7% Latino, 5% Mixed or other. Participants were paid $10.00/hour for taking part in each of two one-hour laboratory sessions and spending approximately two hours completing a Daily Challenge Questionnaire (DCQ; detailed below). Importantly, our sample consisted of adults of three age groups (see Table 1). Preliminary analyses of demographic responses revealed that the age groups did not differ significantly in levels of formal education or perceived health (ANOVA *p*’s > 0.10).

**Table 1:** Mean and (SD) for young, middle-aged, and older adults when computing demographic information, cognitive assessments of intelligence, and average of total number of safe and effective solutions.

	Younger adults (18–29 years old) *N* = 26	Middle-aged adults (30–59 years old) *N* = 32	Older adults (60 + years old) *N* = 27
	
	*M* (SD)	*M* (SD)	*M* (SD)
Age	22.55 (2.14)	46.41 (8.58)	65.78 (6.07)
Years of education	16.81 (2.14)	15.94 (4.53)	15.58 (3.50)
Vocabulary scores (max = 67)	36.94 (10.84)	39.30 (12.45)	37.23 (15.22)
Culture fair test scores (max = 44)	35.58 (5.95)	30.06 (7.37)	23.30 (6.96)
Solutions to younger adult idiographic everyday problems	5.00 (2.51)	4.05 (1.57)	4.02 (1.64)
Solutions to middle-aged adult idiographic everyday problems	4.23 (1.42)	5.39 (2.68)	4.24 (1.55)
Solutions to older adult idiographic everyday problems	3.92 (1.74)	4.58 (1.75)	5.17 (2.32)

#### Procedure: Phase 1

After responding to recruiting materials, participants received a description of study procedures via telephone. Those who chose to take part in the full study next received, by mail, a packet containing introductory instructions including a consent procedure, the DCQ (see below), a demographic questionnaire, and a stamped return envelope.^2^

##### Daily challenge questionnaire

In the central procedure of Phase 1, the DCQ, participants described challenges or problems they faced in everyday life. The descriptions were provided through an open-ended assessment process in which people described their challenges in their own words; this process was inherently sensitive to idiosyncratic content in individual experiences.

Daily challenge questionnaire instructions defined a “challenge” as a difficulty (either isolated or recurrent) that was relatively time consuming (e.g., automobile break-down) or emotional taxing (e.g., arguments with significant other). Challenges were differentiated from minor hassles (e.g., a broken shoe lace), events beyond one’s control (e.g., bad weather), and minor annoyances (e.g., restaurant out of your favorite food). For each challenge provided by a participant, the DCQ contained nine items that guided participants in creating a narrative of the event; for example, participants recounted how they encountered the challenge, whether and how they tried to solve it, what happened consequently, and their perceptions of the challenge (e.g., why it was important or not to solve). Participants then rated difficulty of the challenge (e.g., “How much effort would it take to overcome the challenge completely?”) On 10-point scale, and its openness to solution (“How many possible ways do you think there could be to overcome the challenge?”) on a 5-point scale. These ratings facilitated the identification of challenges whose difficulty and solution openness were similar to those of everyday problems used previously in the literature (Marsiske and Willis, 1995; Pezzuti et al., 2014).

Participants retained the DCQ for up to two weeks. They were asked to complete DCQ responses on each successive day on which they experienced a new challenge, that is, one not described on a prior day. Once identified, these problems were incorporated into the problem sets employed in Phase 2. Importantly, we did not ask to participants to also write solutions when identifying the problems in their diary, that is, to avoid practice effects during the laboratory assessment of their problem solving ability. Also, to avoid recent recollection of the problem from their diary we schedule the laboratory session at least 7–10 days after the DCQ was received, with the average time spent in between sessions estimated around two weeks.^3^

##### Design and matching procedure for problem sets

Our goal was to evaluate any given individual’s performance on three types of everyday problems: ones drawn from the individual’s own experience and from the experiences of two other individuals representing each of the two other age groups. We accomplished this goal through a matching procedure. Subsequent to completion of the DCQ, and prior to participation in Phase 2, the research team created for each individual participant a six-item problem set to be attempted in the Phase 2 laboratory session. Of the six problems, two came from that individual’s DCQ, and the other four came from DCQ responses of participants from the other two other age groups, that is, the two age groups other than that of the given participant. The research team endeavored to identifying problems that were substantively distinct from one another, similar in perceived difficulty, and in all cases were perceived to be open to multiple solutions. In the language of experimental design, the matching procedure yielded a 3-×-3 mixed between-within subjects design.^4^

Analyses indicated that, as desired, younger, middle-aged, and older-adult problem did not differ in perceived difficulty or in openness to multiple solutions, ANOVA p’s. > 0.10. For all groups, mean difficulty ratings were in the range of 6.3–7.0, and openness ratings were in the range of 3.2–3.6.

#### Procedure: Phase Two

Participants were asked to attend two one-hour laboratory sessions. The two sessions were a week apart and followed a random order of presentation. Specifically, 43 participants started with session 1, while 42 participants started with session 2. Results indicated that session order did not affect the total number of safe and effective solutions participant provided, across all age groups, (ANOVA p’s. > 0.10).

##### Session 1

During the first session, an overview of the study was presented verbally and informed consent procedures were conducted. Everyday problem solving ability subsequently was assessed using a procedure adapted from Denney and Palmer (1981; also see Haught et al., 2000; Pezzuti et al., 2014). Participants were asked to generate as many solutions as possible to each of six everyday problems in the matched-participants problem set. Problems were presented in a distinct random order for each participant.

For each problem, short problem descriptions were presented in writing and orally. Participants then were asked to (a) describe what they “perceive to be the optimal solution to the problem,” (b) “discuss as many solutions as possible that they themselves would consider doing,” and (c) “discuss as many solutions as possible that they themselves would not attempt but others might do.” The third prompt was included because older adults, due to their considerable life experience, may discount solutions they believe are inefficacious (Denney et al., 1992; Berg et al., 1998b). Instructions emphasized that the problems do not have singularly correct or incorrect responses and that they could take as much time as they wish to respond. Participants responded orally; sessions were sound recorded.

#### Total Number of Safe and Effective Solutions

The main dependent variable was the total number of safe and effective solutions provided by each participant. Such solutions were identified following three criteria introduced by Denney and Pearce (1989) and later clarified by Marsiske and Willis (1995).

(1) To qualify as a safe and effective solution, each solution must pass a “relevance test.” A relevant solution is one that targets the problem at its root rather than addressing merely a fleeting aspect of it, or missing the problem entirely. For instance “if a middle-aged man is saddened by the lack of attention received from his partner” a solution is not “trying to fix his car” or “go on a diet”– these, as formulated, do not resolve the problem at hand.

(2) Safe solutions are attempts at solving a problem without violating safety or social norms for all people and objects involved. For instance, “if a young couple is bothered by a leaky faucet at their rental apartment” a solution is not breaking the entire pipe underneath the faucet (so that the landlord can be more motivated to fix it), but instead would be to either ask the landlord to look into it or having the problem addressed by a plumber. Or, if the problem is “make more money” a solution would not be “robbing a bank.” (Empirically, participants in our sample provided zero non-safe solutions.)

(3) Effective solutions are one that addresses problems in both short and long term scenarios. If the problem is “a person wants to increase social contact after a break-up,” a solution could be “participate in group activities (book club, or team-sports) one enjoys to meet kindred spirit individuals” – so that in the short term one might address their loneliness, while in the long term, also create stable social contacts. A solution such as “feel free to be sad” might be useful in the short term but it would not work in the long term.

In addition, we did not allow for redundancy of safe and effective solutions, that is, we did not count safe and effective solutions twice. So in the example above in number 3, a solution “joining a group activity a person might like to meet more like minded individuals” was counted only as one solution even if the problem solver also said “joining a church group if the person is religious” or “consider joining a sport group if they are into sports.” This is because the latter two instances are specifications of the former solution. If, however, a general strategy was proposed that could create multiple independent solutions, we counted only the multiple independent solutions and not the general strategy. For instance, for the problem “someone is new to town and they are struggling to make new friends” a general strategy such as “go out more often” would not be counted as one solution if, in the same sentence, the participant specified independent solutions that as “go to the weekly happy hours with colleagues,” “go more often to community events to meet your neighbors,” or “go to a bar and try to strike up a conversation with someone seating next to you.”

Two independent coders, blind to the hypothesis and to problems’ type, were trained on the above criteria. They listened to the audio recordings and identified each safe and effective solution. Inter-coder reliability was assessed at 90%; disagreements were resolved via discussion.

##### Session 2

Remaining measures were administered in a separate study session to avoid fatigue effects. Participants completed measures of several factors that may influence everyday problem solving, including vocabulary (Thurstone and Thurstone, 1962) and fluid intelligence via the Culture Fair Test (Cattell et al., 1941).

## Results

### Preliminary Analyses

Before turning to our main hypotheses, we analyzed the content of the problems that participants described when completing the DCQ. This content analysis revealed that the problems selected from the DCQ for use in Phase 2 of the study presented challenges that were primarily in relation to people. Of the 180 problems selected, 82% were purely interpersonal; with the remaining being instrumental problems, albeit not completely free from interpersonal influences (see Appendix, for a sample of problems in the study).

Vocabulary and fluid intelligence measures were correlated with participants’ age. Fluid intelligence and chronological age were negatively correlated [*r* (83) = −0.63, *p* < 0.01], whereas vocabulary and chronological age were positively correlated [*r* (83) = 0.35, *p* < 0.01]. Both measures of vocabulary [*r* (83) = 0.41, *p* < 0.01] and fluid intelligence [*r* (83) = 0.31, *p* < 0.01] were positively correlated with the number of safe and effective solutions generated – but when we used them as covariates, they did not change the statistical significance of the main results described below (for more descriptive statistics please cf. Table 1).

### Everyday Problem Solving Performance

**FIGURE 1:**
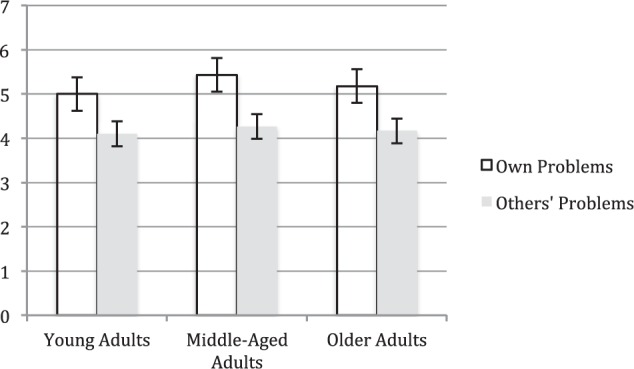
Average of total number of safe and effective solutions (on the *Y* axis) to own and others’ everyday problems provided by younger, middle-aged, and older adults (on the *X* axis).

Results bearing on our main hypothesis, that individuals would perform at highest levels when confronting idiographically-identified problems of maximal relevance to them (“own problems”), are displayed in Figure 1. As shown, problem-solving performance varied across problem context in the anticipated manner each of our three participant groups. Younger Adults, Middle-Aged Adults, and Older Adults each generated more safe and effective solutions to idiographically-identified “own” problems than to “others” problems. We tested our main hypothesis using a repeated-measures MANOVA in which type of problem (own; others’) was a within-person factor and participant age group (young, middle-aged, older adult) was a between-person factor. This analysis confirmed that the number of safe and effective solutions participants generated varied across problems the two types (own; others’), with participants displaying significantly better performance on own problems as compared to others’ problems *F*(1,82) = 28.74, *p* < 0.01, $\eta_{p}^{2}$ = 0.26.

We tested our second set of hypotheses using a repeated measures-MANOVA whose within-person factor was type of problem (young adult problems, middle-aged adult problems, and older adult problems) and whose between-person factor was participant group (young, middle-aged, and older adults).

Again within-subjects analysis indicated that younger adults [*F*(2,24) = 7.87, *p* < 0.01, $\eta_{p}^{2}$ = 0.40], middle-aged adults [*F*(2,30) = 8.56, *p* < 0.01, $\eta_{p}^{2}$ = 0.36], and older adults [*F*(2,25) = 4.04, *p* < 0.05, $\eta_{p}^{2}$ = 0.24] each performed at significantly higher levels on their own problems than on problems from other age groups (see Table 1 for means and standard deviations).

The results also yielded a significant interaction between three different types of problems per different age groups, *F*(4,164) = 9.57, *p* < 0.001, $\eta_{p}^{2}$ = 0.19. The interaction was driven by the fact that middle-aged adults proposed a significantly a higher total number of safe and effective solutions than younger adults and older adults (quadratic contrast effect = −0.93, *SE* = 0.37, *p* < 0.01, $\eta_{p}^{2}$ = 0.07) regarding middle-aged adult problems. Also older adults proposed more safe and effective solutions than middle-aged and younger adults regarding older adult problems (linear contrast effect = 0.88 *SE* = 0.377, *p* < 0.05, *d* = 0.61 with younger adult group). Younger adults only marginally proposed more safe and effective solutions than older and middle-aged adults (linear contrast effect = −0.69, *SE* = 0.38, *p* = 0.067, *d* = 0.46 with older adult group) on the younger adult problems.

## Discussion

The results were consistent with the overarching idea that motivated this research. We hypothesized that people’s ability to solve everyday problems would vary contextually; specifically, we expected that individuals of all ages would display higher problem-solving performance when confronting problems of maximal ecological relevance to them. These problems were identified on an idiographic basis through use of a DCQ. The problems reported were mostly interpersonal. As anticipated, findings revealed a highly significant effect of problem type, with participants as a whole displaying significantly higher levels of performance on personally-relevant problems identified idiographically.

This directional pattern of results, in which “own problem” performance was overall better, held for each of our three participant groups: Younger, Middle-Aged, and Older Adults. The difference between own-problem and other-problem performance levels was found to be significant for two of the groups considered separately, Middle-Aged and Older Adults, and was marginally significant for the third, Younger Adults. We cannot rule out the fact that this marginal effect is due to our small sample size, which is certainly a limitation of this study. Specifically, the analysis of the effect size for the between groups comparison would suggest that with a larger sample of participants significant age differences could be fully achieved on the younger adult problems as well.

As noted initially, the idiographic methods employed inherently relinquish experimental control; in fact any given individual’s maximally-relevant problems may vary from another’s in multiple ways. To address this issue, in the selection of own problems, we controlled for perceived importance, effort, openness to solution, number of attempts in the past, etc.

Perhaps most importantly, the “own problems” selected here could have the advantage of being already familiar to problem solvers. Idiographically captured expertise in interpersonal problem solving cannot be teased apart from having experienced day-to-day problems beforehand. The more people would have familiarity with everyday problems the better their everyday problem-solving skills. In the language originally introduced by Simon (1973) and Reitman (1964), thinking about the problem would facilitate the exploration of the problem solving space. If participants already thought about the problem when reporting it in their DCQ, they might have already explored the problem solving space of the given problem. Similarly, experts do better than non-experts because they practice more, have thought about the problem endless times before, and also retain a higher ability to solve novel problems within their domain of expertise.

We deliberately accepted this loss of experimental control in order to take the valuable step of maximizing the personal relevance of everyday problems, specifically in the interpersonal domain. In fact, there is a gap in the literature concerning the assessment of cognitive performance using tailored assessment materials to better capture whether older adults use expertise developed in familiar contexts to maintain their everyday problem-solving ability.

To illustrate, consider a well-known anecdotal example provided by Baltes and Baltes (1990): the pianist Arthur Rubenstein maintained extraordinarily high levels of artistic performance by employing domain-specific expertise through which he compensated for age-related declines in motoric speed and flexibility. A psychological assessment of Rubenstein’s cognitive capacities ideally would have revealed this capability. Yet, consider what would have happened if Rubenstein had participated in a traditional study employing nomothetic assessments featuring a fixed set of everyday problems. Since concert-level piano performance is a rare capability, it is unlikely to be featured in any nomothetic assessment device. Nomothetic assessment might have completely overlooked Rubenstein’s unique abilities. In contrast, a person-focused strategy that identifies domains of particular relevance to the individual case might have succeeded in identifying Rubenstein’s key domain of expertise, and thus more fully captured his performance capacities. Such a person-focused strategy, as well as the specific pattern of results that was uncovered through use of such a strategy in the present study, are consistent with a contextual perspective on individual performance and well-being across the life course (Berg et al., 1998a; Strough and Keener, 2014).

Previous research also suggests that everyday experience among older adults might reveal a different pattern of results than just typically age-related declines. Studies involving older adults who possessed specific domain expertise such as typists (Salthouse, 1984) or pilots (Morrow et al., 2001) proved this point. When performing expertise-relevant tasks, then, older adults have been found to perform at a level equal to that of younger adults, even though they may have lesser cognitive processing abilities than younger participants (see Thornton and Dumke, 2005 for a meta-analysis).

The scope of our work is of course limited to everyday problem solving abilities. Thus, its implications cannot immediately be translated more broadly into the domain of expertise. A potential application of personal everyday expertise, however, could be used to chart an intervention. Suppose an intervention program was aimed at increasing physical activity among adults of different age groups. New findings indicate that blocks to engaging in physical activity go beyond schedule constraints, vary between people, and are uniquely personal (Artistico et al., 2013).

“My problems are solvable,” this paper’s title, could be viewed broadly as a mind set that shapes experience and action beyond just the capacity to generate problem solutions. People might feel more empowered knowing that, despite limitations and cognitive declines, they may maintain the capacity to address the most significant, relevant problems that they experience. This recognition could boost a sense of personal confidence and increase self-efficacy for problem solving. Such positive mindsets may not boost older-adult performance in all circumstances. For example, when problems are novel, their solution may require rapid working-memory processing which, inevitably, declines with age (see Marsiske and Margrett, 2006).

Yet we endorse Nelson (2016) position that older adults will tend to take positive action to promote their well-being “to the degree that [they] have strong internal control beliefs... and disbelieve negative stereotypes” (p. 277) about aging. Interventions designed to influence the degree to which positive and negative age stereotypes are cognitively accessible support this conclusion. Levy et al. (2014) demonstrated that an intervention to activate positive age-related stereotypes boosted older adults’ self-perceptions which, in turn, led to increased physical functioning. In principle, an intervention approach that incorporated idiographic methods to identify problem-solving skills and self-perceptions at the level of the individual case could be even more engaging to older adults and even more efficacious in boosting positive mind sets. Perhaps one could anticipate a transfer of expertise from resolved problems to unique problems (i.e., exercising more) not yet resolved. Understanding the determinants and therefore their individualized variations in performance across contexts may facilitate the design of tailored interventions to boost self-perceptions, motivation, and resilience in important life domains in which older adults may lack prior experiences of personal mastery.

## Author Contributions

DA and DC designed the research and provided several drafts of writing to reach the final stage of this manuscript. CG helped analyzing the data and provided several drafts of writing to reach the final stage of this manuscript.

## Conflict of Interest Statement

The authors declare that the research was conducted in the absence of any commercial or financial relationships that could be construed as a potential conflict of interest.

## Footnotes

1 Our research revealed that (Artistico et al., 2003, 2010), averaged across participants, the interaction problem type x participants’ age group has a robust effect on everyday problem solving (*R*^2^ = 0.20). Given an effect of this magnitude, we calculated that cell in the range of 25–30 cases should be detecting an effect considering an alpha of 0.05 and a desired power of 0.80. Our actual interaction effect produced a power of 0.97.

2 One participant elected to complete these measures orally, over the phone – no differences were reported in their results.

3 A precise log of the exact number of days was not kept in the records – this is a limitation of our study procedure.

4 During Phase 2, participants also attempted additional problems, in the domains of loneliness and finance, results for these are reported elsewhere (Artistico et al., 2010). These data and results discussed here are completely novel.

## Appendix

### Three Examples of Socio-Emotional or Interpersonal Problems

(1) A friend criticizes you for an important decision that you made. You find that your friend is being judgmental and unsupportive, this hurts you because you trust this person and expect them to be there for you and support you.
(2) You are doing something you know perfectly well how to do by yourself, and someone begins giving you advice you neither need nor want. The person is trying to make you seem like you do not know what you are doing when in fact you are certain you are right.
(3) You feel like your parents or children do not have enough time to spend with you.

You understand that they are busy with work, school and their own personal lives but, you feel neglected and think they could make time for you if they really wanted to.

### Three Examples of Instrumental Problems

(1) Suppose your health insurance does not cover the medications that your doctor has prescribed. You urgently need these medications but the price is too high and your insurance says these medications are not eligible for coverage.
(2) You have let your home become too cluttered with items you use infrequently but which have much sentimental value for you. You have considered giving some items away but they mean too much to you, at the same time your home is now uncomfortable and you cannot find space anywhere.
(3) You are trying to find an item of clothing in a style you like, but you are unable to find it after shopping for several hours. You have wanted to purchase this particular item for a long time and set that day aside specifically to find that item.
